# Evaluation of bone metastatic burden by bone SPECT/CT in metastatic prostate cancer patients: defining threshold value for total bone uptake and assessment in radium-223 treated patients

**DOI:** 10.1007/s12149-017-1224-x

**Published:** 2017-12-14

**Authors:** Takuro Umeda, Mitsuru Koizumi, Shohei Fukai, Noriaki Miyaji, Kazuki Motegi, Shuto Nakazawa, Tomohiro Takiguchi

**Affiliations:** Department of Nuclear Medicine, Cancer Institute Hospital, 3-8-11 Ariake, Koto-ku, Tokyo, 135-8550 Japan

**Keywords:** Bone SPECT/CT, TBU, Prostate cancer, Ra-therapy

## Abstract

**Objectives:**

To establish a new three-dimensional quantitative evaluation method for bone metastasis, we applied bone single photon emission tomography with computed tomography (SPECT/CT). The total bone uptake (TBU), which measures active bone metastatic burden, was calculated as the sum of [mean uptake obtained as standardized uptake value (SUV) above a cut-off level] × (the volume of the lesion) in the trunk using bone SPECT/CT. We studied the threshold value and utility of TBU in prostate cancer patients treated with radium-223 (Ra-223) therapy.

**Methods:**

To establish the threshold value of TBU, we compared bone metastatic and non-metastatic regions in 61 prostate cancer patients with bone metastasis and 69 without. Five fixed sites in each patient were selected as evaluation points and divided into bone metastatic and non-metastatic sites. Sensitivity and specificity analysis was applied to establish the threshold level. Using the obtained threshold value, we then calculated the TBU in nine prostate cancer patients who received Ra-223 therapy, and compared the results with the bone scan index (BSI) by BONENAVI^®^ and visual evaluation of bone scintigraphy.

**Results:**

Uptake was significantly lower in non-metastatic sites in patients with bone metastasis than in patients without metastasis. Sensitivity and specificity analysis revealed SUV = 7.0 as the threshold level. There was a discrepancy between TBU and BSI change in two of the nine patients, in whom TBU change correlated with visual judgement, but BSI change did not. In two patients, BSI was nearly 0 throughout the course, but the TBU was positive and changed, although the change was not large. These results suggest that TBU may be more accurate and sensitive than BSI for quantitative evaluation of active bone metastatic burden.

**Conclusion:**

We established a threshold value (SUV > 7.0) for three-dimensional TBU for evaluating active bone metastatic burden in prostate cancer patients using bone SPECT/CT. Despite the small number of patients, we expect the change in TBU could be more accurate and sensitive than the change in BSI among patients who received Ra-223.

## Introduction

Bone metastasis from prostate cancer is often osteoblastic. Blastic bone metastases have been regarded as unmeasurable [1]. Several methods of measuring the bone metastatic burden have been reported. Visual evaluation of bone scintigraphy (BS) was proposed by Soloway as the extent of disease (EOD) [2]. This EOD system has been used for a long time and is still in common use. BONENAVI^®^ (EXINI Bone^®^) using whole-body planar bone scintigraphy and artificial intelligence (AI) has also been proposed [3–5]. This BONENAVI^®^ uses two automatic indices: artificial neural network (ANN) as the probability index for bone metastasis, and the bone scan index (BSI) as the degree of bone tumor burden [6]. Several publications have been reported the usefulness of BSI for evaluating the effects of therapy for bone metastasis in metastatic prostate cancer patients [7–9]. BSI is becoming a recognized method for quantifying bone tumor burden [10].

BSI is obtained from the information in planar images, that is, two-dimensional information. Etchebehere et al. published a three-dimensional method using fluoride positron emission tomography and computed tomography (PET/CT) to evaluate active bone metastatic burden [11], using a tentative threshold of standardized uptake value (SUV) of 10.0 in fluoride PET/CT. The index derived from the product of mean SUV × voxel of interest (VOI)_10_, named TLF_10_, had the potential to predict therapeutic response [11, 12]. Quantitative measuring of radionuclide uptake using single photon computer tomography with X-ray computed tomography (SPECT/CT) can now be used to calculate the SUV [13–16]. Using this technique, we have begun to evaluate the bone metastatic burden from the thorax to pelvis using three-dimensional bone SPECT/CT.

In the present study, we aimed to determine the essential factor SUV cut-off level for bone SPECT/CT for evaluating the degree of radionuclide uptake in bone metastasis in patients with prostate cancer. We then applied this technique to metastatic prostate cancer patients receiving radium (Ra)-223 therapy [17], and compared the utility of this technique with BSI. To our knowledge, this is the first investigation to evaluate bone metastatic burden using bone SPECT/CT in metastatic prostate cancer patients treated with radium-223.

## Patients and methods

### Patients

Patients with histologically confirmed prostate cancer who underwent bone SPECT/CT from 1 April 2016 to 31 March 2017 were enrolled in this retrospective clinical study. The study was approved by our institutional ethics committee (no. 2016-1140).

The inclusion criteria were histologically proven prostate cancer and bone SPECT/CT from the lower thorax through to the proximal portion of the femur. These patients were divided into patients with and without bone metastasis.

### BS and bone SPECT/CT

BS was performed approximately 3–4 h after intravenous injection of 740 MBq technetium-99m methylene diphosphonate (^99m^Tc-MDP, Fujifilm RI Pharma Co. Ltd., Tokyo, Japan). Whole-body images were obtained using a gamma camera (Symbia Intevo, Siemens Healthcare, Tokyo, Japan). Bone SPECT/CT was performed after taking whole-body images.

SPECT scans were acquired using low-energy high-resolution collimation, a 256 × 256 matrix of 2.4-mm pixel size, and a total of 120 projections over 360° with a duration of 10 s/view. CT scans were performed with 130 kV and 80 mAs using adaptive dose modulation (CARE Dose 4D). The CT data were reconstructed with 3-mm slice thickness using medium sharp and attenuation kernels (B50s and B31s, respectively).

Quantitative SPECT images were reconstructed using a Siemens “xSPECT Quant” [18, 19], with an ordered subset conjugate gradient maximization (OSCGM) algorithm with one subset, thirty iterations, and 6 mm gaussian-filter.

### Selection of non-metastatic or metastatic bone lesions

In patients without bone metastasis whose bone CT and BS were both normal, each five points were regarded as non-metastatic lesions. In patients with bone metastasis, lesions with both abnormal CT (osteoblastic change in the bone) and increased radionuclide uptake were regarded as metastatic lesions, and lesions with normal or non-metastatic CT appearance (normal appearance or sclerotic change in articular surface) and normal radionuclide uptake were regarded as non-metastatic. For example, a patient with rib osseous metastasis only had five non-metastatic lesions, while another patient with ilium metastasis had a metastatic ilium lesion and four other non-metastatic lesions.

### Analysis of bone SPECT/CT

**Fig. 1:**
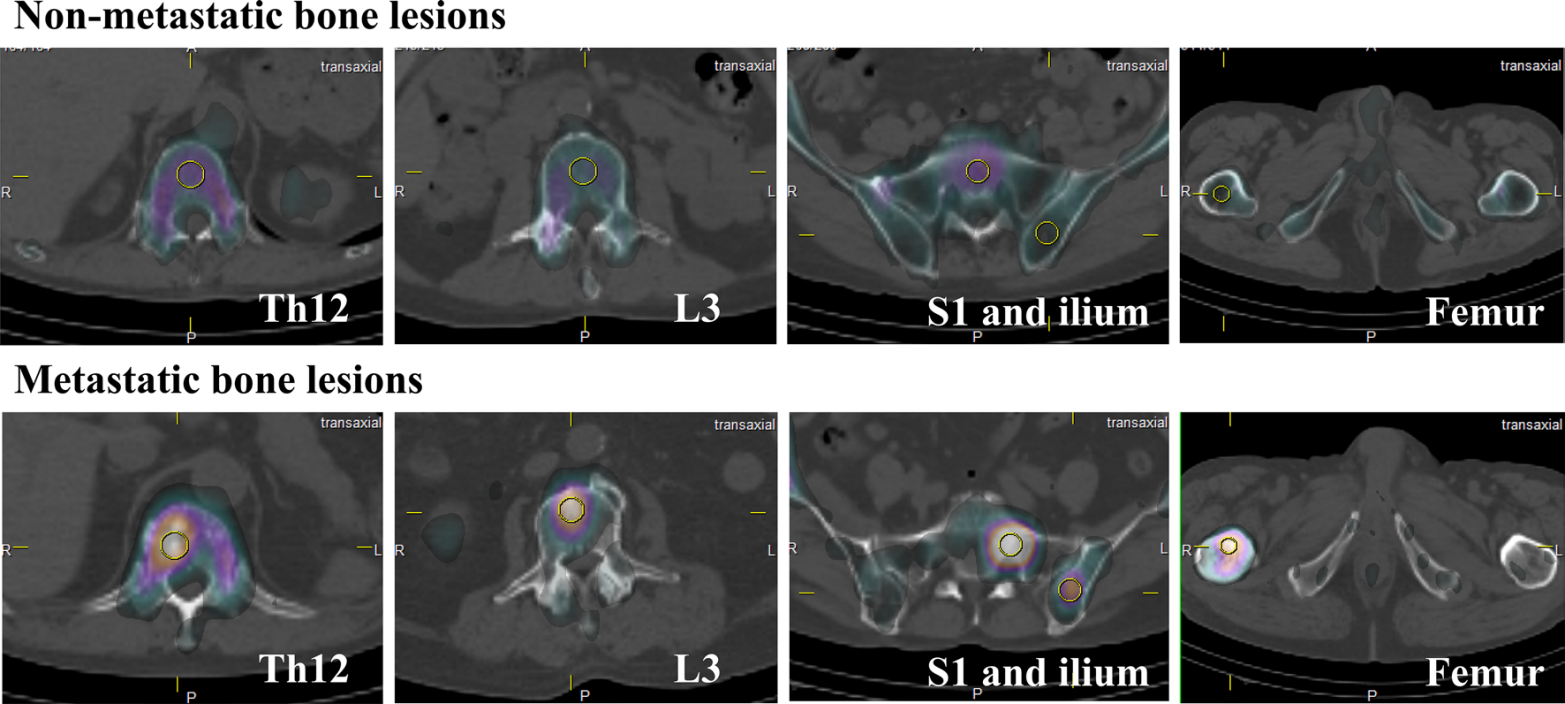
Shows examples of spherical voxel of interest (VOI) placement in the 12th thoracic vertebra, third lumbar vertebra, sacrum, ilium, and femur neck in non-metastatic sites (upper rows) and metastatic sites (lower rows)

In each patient, spherical voxels of interest (VOIs) of 1.42 cm^3^ were placed on five reference points: the 12th thoracic vertebra (Th12), third lumbar vertebra (L3), upper part of the sacrum (S1), the ilium, and the femur neck, and the SUV were measured. The VOI size was same for both non-metastatic and metastatic lesions. As shown in Fig. 1, the VOIs in metastatic lesions were placed at the center of increased radioactivity.

### Total bone uptake (TBU)

Total bone uptake (TBU) calculated using bone SPECT/CT images (from the top of the thorax to the bottom of the pelvis) is considered to be an index of active bone metastatic burden. TBU was obtained using a software, GI-Bone^®^ (Nippon Medi-Physics, Tokyo, Japan).

**Fig. 2:**
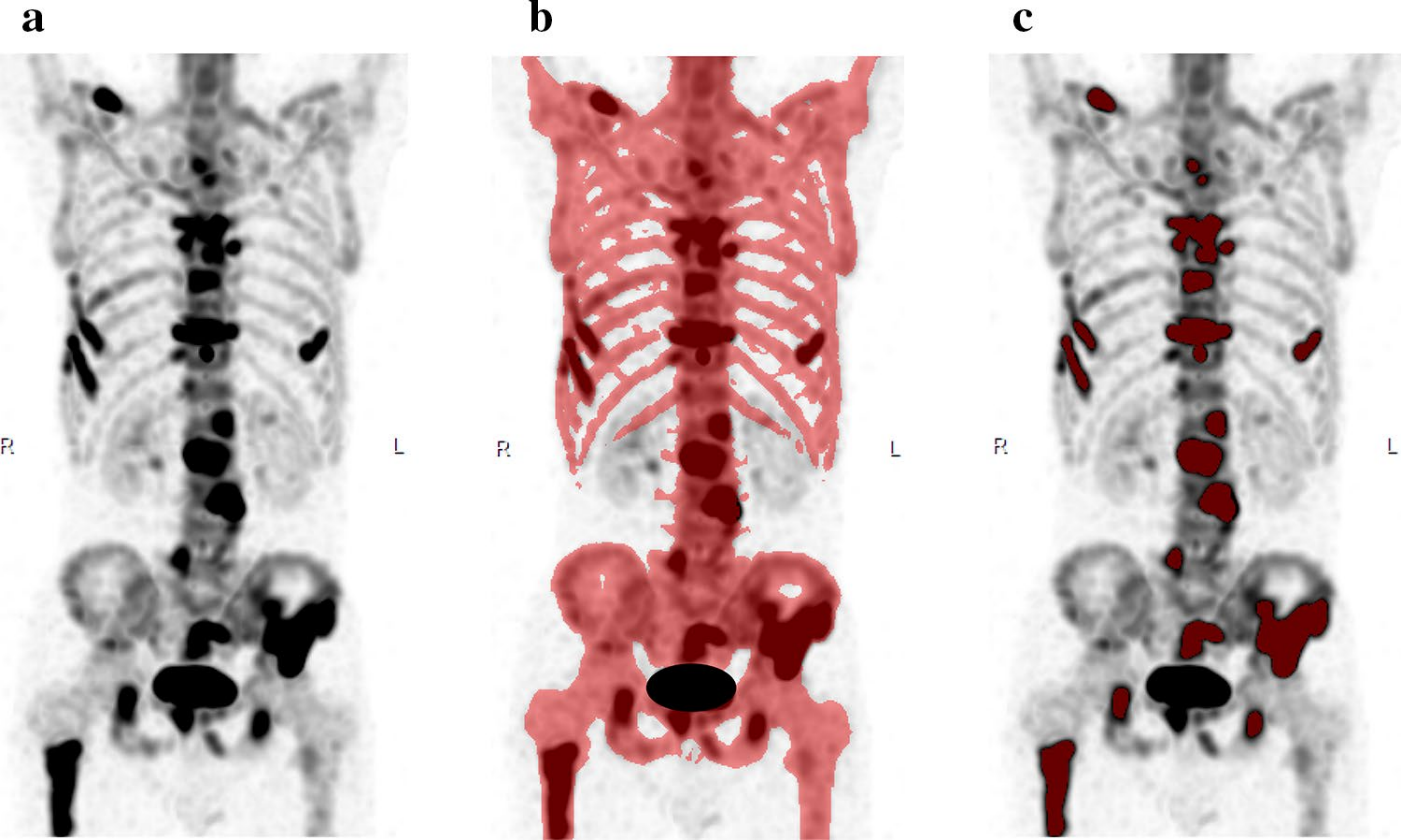
The process of determining total bone uptake (TBU). A maximum intensity projection (MIP) image was selected to explain. MIP image of the trunk was obtained by bone SPECT/CT study (**a**). Bone image (red) was obtained by subtracting non-bone components using 152 CT Hounsfield units (**b**). Areas above SUV = 7.0 was considered as the active bone metastatic burden (red in **c**). Then, TBU was obtained as the sum of (mean SUV above SUV = 7.0) × (volume above SUV = 7.0) for each lesion

TBU was calculated in technetium-99m bone SPECT/CT as follows. Bones were selected on the CT image using a threshold of > 152 Hounsfield units [20], removing all non-osseous volumes (e.g., kidney, bladder, and soft tissues) on the maximum intensity projection (MIP) image. Sections or volumes above a certain radioactivity threshold (obtained in this study) were then selected as active bone metastatic regions. Then, the mean of the regional SUV (SUVmean) above threshold multiplied the regional volume above threshold. The sum of regional (SUVmean above threshold) × (volume above threshold) was regarded as TBU; an index of active bone metastatic burden. In the process, the sum of the volumes above the threshold was obtained as metabolic bone volume (MBV). The process of calculating TBU is shown in Fig. 2.

### SUV threshold for measuring active bone metastatic burden

The maximum of SUV (SUVmax) values of bone metastatic sites and non-metastatic sites were plotted as a sensitivity–specificity curve. The cut-off point was decided based on the minimum distance from the left upper point (0,1) of the receiver operating characteristic (ROC) curve and Youden’s index [21]. We considered the specificity to be more important than sensitivity in this analysis.

We also compared non-metastatic SUVmax values between patients with and without bone metastasis. Because intense uptake by bone metastatic lesions may reduce the uptake by non-metastatic lesions in patients with bone metastasis, radionuclide uptake by non-metastatic bone in bone metastasis patients might be lower than that in normal bone in patients without bone metastasis.

### TBU in patients receiving Ra-223 therapy for bone metastasis

Patients who received Ra-223 therapy (55 KBq/body weight × 6 times, 4-week intervals) were analyzed by bone scintigraphy with SPECT/CT and BSI, and the results were compared with their clinical course. These patients were scheduled to receive bone SPECT/CT before therapy, after three infusions of Ra-223, after six infusions of Ra-223, and at follow-up study if possible. BONENAVI^®^ analysis was also performed, and the changes in TBU and BSI were compared with visual BS judgement.

### Statistical analysis

The cut-off point was decided based on the minimum distance from the (0,1) point of the ROC curve: Min{(1 − sensitivity)^2^ + (1 − specificity)^2^}, and the Youden's index: Max{sensitivity + specificity − 1}.

The mean difference was analyzed using an unpaired Student’s *t* test with Levence test for equality of variances. Statistical analysis was performed using SPSS version 24 (IBM Corp., Armonk, NY, USA). A *p* value < 0.05 was considered to indicate statistical significance.

## Results

### Patients

Sixty-one prostate cancer patients with bone metastasis (age: median 74 years, range 42–87 years) and 69 without bone metastasis (age: median 70 years, range 50–91 years) were analyzed to determine the cut-off level. Among those patients, 47 patients with bone metastasis and 15 patients without bone metastasis were castration-resistant.

Nine patients received Ra-223 therapy (55 kBq/body weight × 6 times, 4-week intervals). Of them, six patients completed six infusions of Ra-223 and three patients who did not complete the course for various reasons.

### SUVmax values in non-metastatic regions

Table 1 shows the SUVmax values in non-metastatic regions in patients with and without bone metastasis. The mean SUVmax values for non-metastatic sites in patients with bone metastasis were significantly lower than those in non-metastatic patients in Th12 (*p* < 0.0001), L3 (*p* = 0.006), the sacrum (*p* < 0.0001), and the ilium (*p* = 0.048). In proximal femur, the mean SUVmax value was lower in metastatic patients than in non-metastatic patients, but there was no statistical significance. Radionuclide uptake by non-metastatic bone was lower in patients with bone metastasis compared with those without bone metastasis.

**Table 1:** Radionuclide uptake in non-metastatic sites in metastatic and non-metastatic patients

Site	Bone metastasis	Number of lesions	Mean SUVmax	SD	*p* value
Th12	Negative	69	5.71	1.37	< **0.0001**
	Positive	41	4.64	1.19	
L3	Negative	69	5.32	1.4	**0.006**
	Positive	40	4.58	1.24	
S1	Negative	69	5.55	1.56	< **0.0001**
	Positive	40	4.27	1.58	
Ilium	Negative	69	4	1.11	**0.048**
	Positive	29	3.53	0.97	
Femur	Negative	69	2.36	0.79	0.231
	Positive	49	2.19	0.73	

Bold values show statistically significant differences

SUVmax maximum standardized uptake value

### Sensitivity–specificity curve

**Fig. 3:**
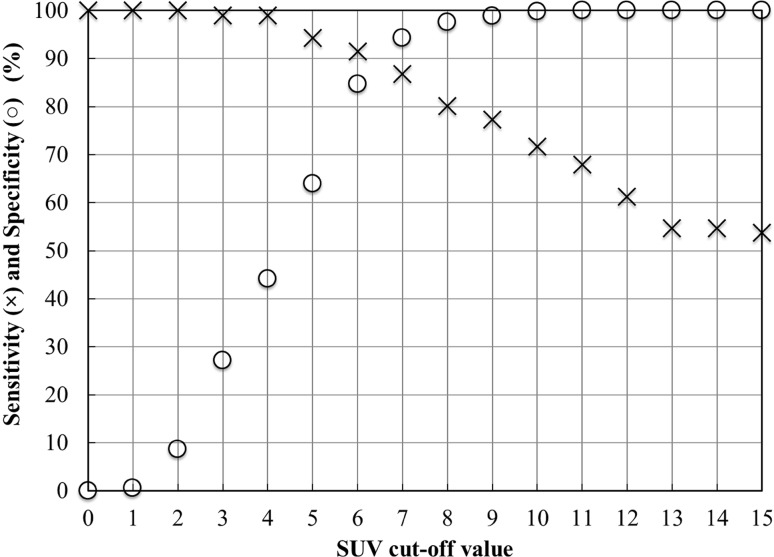
Sensitivity (multiple sign) and specificity (open circle) curves in relation to SUV threshold change. The two curves crossed between SUV 6 and 7

Figure 3 shows the sensitivity–specificity curves. We used the non-metastatic values for patients with and without bone metastasis. The sensitivity and specificity, the distance from the left upper point (0,1) of the ROC curve and Youden’s index values are shown in Table 2. The minimum distance was at SUVmax = 7.0, and the Youden’s index was maximal at SUVmax = 7.0. The specificity was 94.3% and the sensitivity was 87.0% at SUVmax = 7.0. We therefore chose an SUV value > 7.0 as indicating active bone metastatic lesions.

**Table 2:** Sensitivity, specificity, and cut-off value

SUV cut-off value	Sensitivity	Specificity	Youden’s index^a^	Distance^b^
0	1	0	0	1
1	1	0.006	0.006	0.99
2	1	0.086	0.086	0.84
3	0.99	0.272	0.263	0.53
4	0.99	0.441	0.432	0.31
5	0.94	0.64	0.583	0.13
6	0.92	0.847	0.763	0.03
**7**	0.87	0.943	**0.811**	**0.02**
8	0.8	0.976	0.778	0.04
9	0.77	0.989	0.763	0.05
10	0.72	0.998	0.715	0.08
11	0.68	1	0.679	0.1
12	0.61	1	0.613	0.15
13	0.55	1	0.547	0.21
14	0.55	1	0.547	0.21
15	0.54	1	0.538	0.21

Bold values show the best values

SUV standardized uptake value

^a^Youden’s index: sensitivity + specificity − 1

^b^Distance from left upper point (0,1) of reciever operating characteristic curve, calculated as (1 − sensitivity)^2^ + (1 − specificity)^2^

### TBU change in patients with Ra-223 therapy

TBU values were calculated using SUV > 7.0 as active bone lesions. Table 3 summarizes changes in TBU, BSI, alkaline phosphatase (Al-p: U/L, reference value 106–322) and prostate-specific antigen (PSA) pre-therapy, during therapy, and post-therapy, Ra-223 therapy status (completed or dropped out), visual BS judgement, and clinical judgement. Six patients completed six Ra-223 infusions, and three patients dropped out.

**Table 3:** Bone scintigraphy (bone SPECT/CT and BONENAVI^®^) in patients with Ra-223 therapy

			Bone scintigraphy (BS)							
			1st	2nd	3rd	4th				
No	Age (year)		Pre-Ra-223	Ra-223 3 cycles	Ra-223 6 cycles		Ra-223 therapy	Visual BS judge	TBU vs BSI	Clinical judgement
						After 5 m				
1	75	TBU	27,189	53,647	38,135	22,565	Completed	Effective	Agreed	Effective
		BSI	0.202	0.402	0.31	0.197		Flare-up		
		PSA (ng/ml)	0.52	0.84	1.29	3.24				
		Al-p (U/L)	157	180	162	161				
2	81	TBU	1092	1276	3017		Completed	Effective	Agreed	PD
		BSI	0.006	0	0					Progression of DM
		PSA (ng/ml)	73.54	114.02	353.89					
		Al-p (U/L)	243	227	235					
3	80	TBU	648,302	526,485	1,548,740		Completed	PD	Agreed	PD
		BSI	1.514	1.53	5.301					Pneumonia, death
		PSA (ng/ml)	9.05	34.44	257.2					
		Al-p (U/L)	233	166	444					
4	71	TBU	104,302	162,677	**255,650**		Completed	PD	**Disagreed**	PD
		BSI	0.136	0.206	**0.082**				**At third BS**	6 months later, hepatic metastasis
		PSA (ng/ml)	294.67	888.61	2990.82					
		Al-p (U/L)	223	250	437					
5	72	TBU	294,029	123,199	97,044		Completed	Effective	Agreed	Effective
		BSI	0.82	0.356	0.182					
		PSA (ng/ml)	8.37	7.98	7.65					
		Al-p (U/L)	128	123	129					
6	65	TBU	51,802	105,553	72,197		Completed	Effective	Agreed	PD
		BSI	0.363	0.535	0.298			Flare-up		Primary lesion and LN metastasis progression
		PSA (ng/ml)	11.14	19.35	48.66					
		Al-p (U/L)	131	125	126					
						After 10 m				
7	83	TBU	14,474	17,092	ND	816	Dropped out after four cycles	Effective	Agreed	PD
		BSI	0	0	ND	0	Due to worsing of PS			PS worsen
		PSA (ng/ml)	17.58	41.19	94.37	70.76				Hepatic metastasis at 10 m after, death at 11 m after
		Al-p (U/L)	231	196	161	451				
8	79	TBU	165,769	**217,451**	ND		Dropped out after five cycles	PD or flare-up	**Disagreed**	PD
		BSI	0.785	**0.774**	ND		Due to neurogic symptom		**At second BS**	Neurogenic symptom progressed
		PSA (ng/ml)	81.56	309.65	1145.64					Para-aortic LN metastssia
		Al-p (U/L)	373	435	1024					
9	73	TBU	384,020	196,676	ND		Dropped out after two cycles	Effective	Agreed	PD
		BSI	4.067	1.287	ND		Due to hepatic metastasis			Hepatic metastasis
		PSA (ng/ml)	120.35	170.96	ND					
		Al-p (U/L)	139	139	ND					

Bold values show the discrepancy between TBU and BSI

TBU total bone uptake, BSI bone scan index (%), PSA prostate-specific antigen (ng/ml), Al-p alkaline phosphatase (U/L; reference value 106–322), Ra radium, BS bone scintigraphy, m month, PD progression of disease, DM diabetes mellitus, LN lymph node, ND not done, PS performance status

**Fig. 4:**
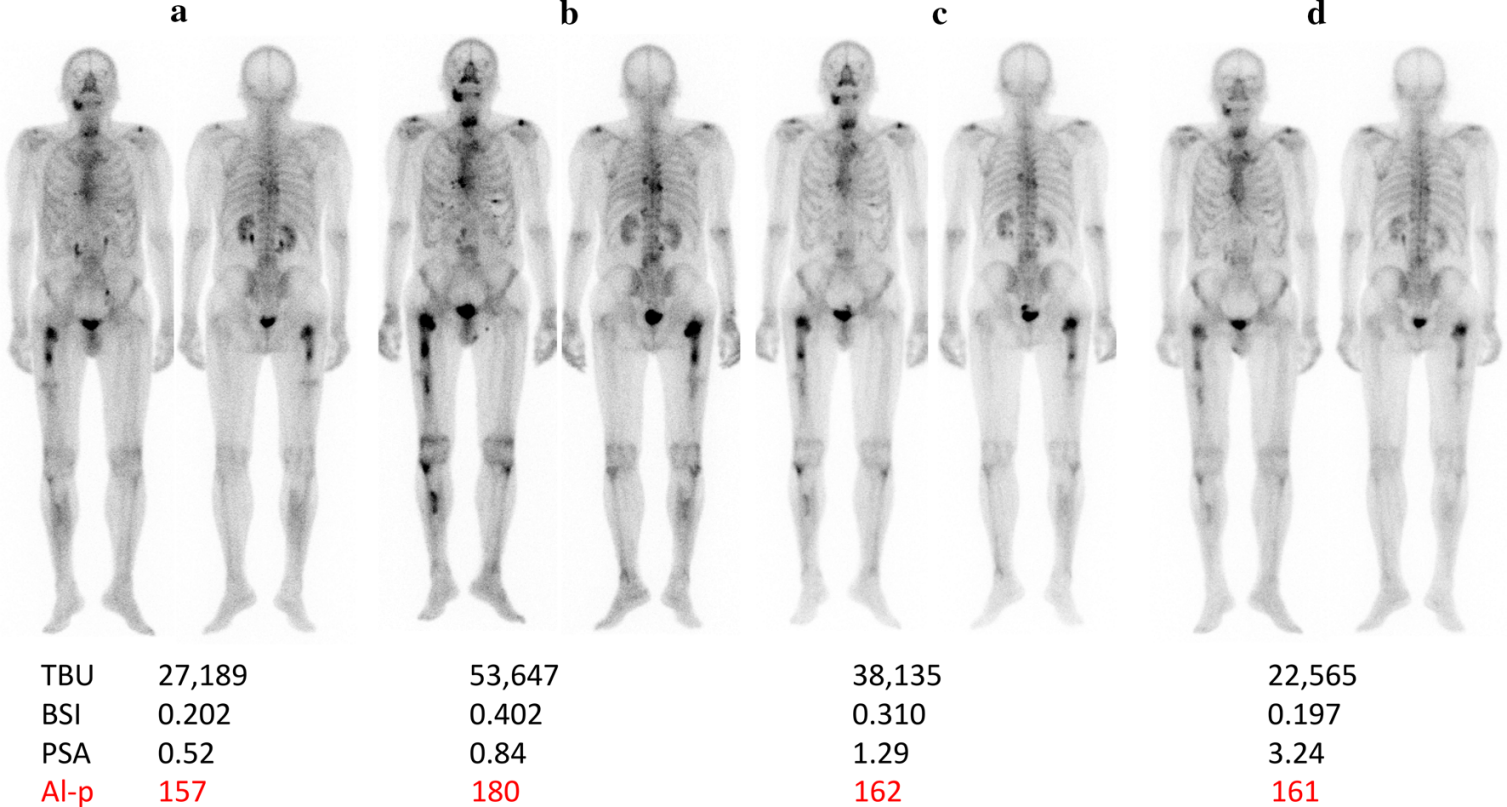
Serial BS of an Ra-223 treated patient (Case 1) were shown: **a** pre-treatment; **b** just after three cycles of Ra-223 therapy; **c** just after six cycles of Ra-223 therapy; **d** 5 month after the completion of Ra-223 therapy (six cycles). In the bottom, TBU, BSI and PSA values at the time were shown. Visual image, TBU and BSI showed the progression or increase in the second image and gradually returned to initial values. PSA change was minimal even though there was a gradual increase; no specific change in symptoms was noticed. The second bone scan was regarded as a BS flare phenomenon. TBU and BSI changes were concordant with and supported the BS flare

Figure 4 shows the results of serial bone scintigraphy (case 1) of pre-Ra-223 therapy, during Ra-223 therapy (after 3 times of Ra-223 infusions), at the end of Ra-223 therapy (after 6 times of Ra-223 infusions), and 5-month after the Ra-223 completion. Bone scan showed increased uptake on the second study (after three infusions), and the uptake then decreased gradually. We judged the second BS as a flare-up. The changes in BSI and TBU supported the BS flare phenomenon.

**Fig. 5:**
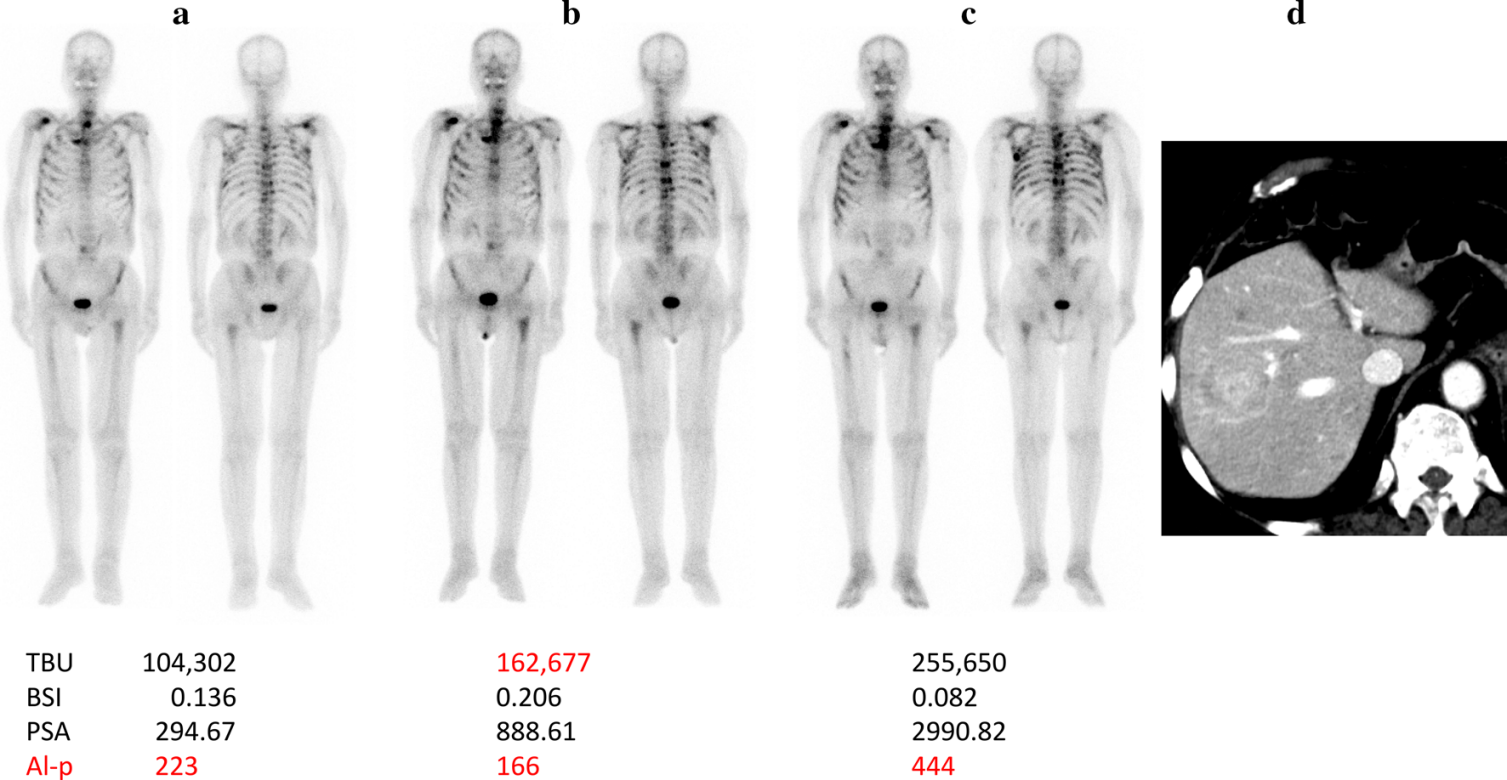
Serial BS of an Ra-223 treated patient (Case 4) were shown: **a** pre-treatment, **b** just after three cycles of Ra-223 therapy, and **c** just after six cycles of Ra-223 therapy. Visual evaluation was judged as indicating progression of bone metastasis. TBU increased constantly and agreed with the visual evaluation, however, BSI change was increased in the second study and decreased in the third study. The change in BSI was not concordant with the visual evaluation, Al-p change, and TBU change. The PSA change increased consistently, supporting progression of the disease

Figure 5 shows serial bone scintigraphy results (Case 4) pre-Ra-223 therapy, during Ra-223 therapy (after three infusions), and at the end of Ra-223 therapy (after six infusions). There were discrepancies between the TBU and BSI changes: TBU increased consistently (104,302–162,677–255,650), but BSI increased from pre-therapy to during therapy, but decreased at the end (0.136–0.206–0.082). PSA, Al-p and visual abnormal uptake on BS increased consistently. In this case, TBU correlated with visual BS and Al-p changes, while BSI did not. Case 4 was the BSI and TBU disagreed patient. Case 8 was another BSI and TBU disagreed patient whose second BS results showed increased uptake on visual evaluation and the Al-p increased on the second. The TBU increased, but the BSI remained unchanged.

In the other seven patients, changes in TBU and BSI were roughly similar. However, in some cases (Cases 2 and 7) whose bone metastatic burden was minute and changes were small, there was no change in BSI, but TBU detected small changes.

## Discussion

Bone metastasis from prostate cancer often shows osteoblastic characteristics. Although osteolytic and mixed types of bone metastasis can be monitored by CT and MRI, osteoblastic metastasis is unmeasurable [1]. However, bone scan index (BSI), a method using artificial intelligence (AI), has recently been developed [3–6] and BSI has been reported as a useful imaging biomarker for evaluating the effect of therapies for osseous metastasis of prostate cancer [7–9].

BSI was obtained from two-dimensional planar bone scintigraphy. We have tried to evaluate the therapeutic effect on osseous metastasis in prostate cancer patients based on three-dimensional bone SPECT/CT images.

We focused on the SUV as an index of uptake value, and regarded active osseous metastatic lesions as those with increased radionuclide uptake. Bone metastatic lesions in the trunk above a certain level of radioactivity were defined as active osseous lesions, and lesion activity was calculated as the volume above a certain level × the mean activity of the lesion: (volume above SUV cut-off level) × (mean SUV). Each patient’s bone metastatic activity, named TBU, was obtained by the sum of (volume above SUV cut-off level) × (mean SUV).

To obtain a certain level, the cut-off value for SUV, prostate cancer patients who underwent bone SPECT/CT were collected. SUV values for five fixed points (Th12, L3, the sacrum, the ilium, and the proximal femur) were compared between patients with and without osseous metastasis. We selected these five points because of these regions were the frequent sites of bone metastasis in prostate cancer. Rib was not selected because it is a thin bone (large VOI cannot place). The SUV cut-off value for discriminating between metastatic and non-metastatic bone was determined according to the sensitivity and the specificity.

In that process, we found that radionuclide uptake in non-metastatic lesions in patients without metastasis was statistically significantly higher than that in metastatic patients. This may be explained that because of intense uptake in osseous metastatic lesions, the uptake by non-metastatic regions was decreased in patients with osseous metastasis.

The SUV cut-off level between metastatic and non-metastatic lesions was SUV = 7.0, based on sensitivity and specificity analysis in prostate cancer patients. The SUV for normal vertebrae has been reported as 5.9 ± 1.5 [16], 5.6 ± 1.9 [22], and 7.1 ± 0.4 [23]. Because the uptake by non-metastatic bone in bone metastasis patients was lower in this study, we considered that a cut-off level of SUV = 7.0 was reasonable.

We compared TBU and BSI in patients who received Ra-223 therapy and found similar changes in seven out of nine patients, but discrepancies in two patients. When compared to visual BS judgements, the change in TBU was more concordant with the visual evaluation than the change in BSI. In these two patients, the change in TBU was consistent with the change in Al-p. Moreover, in other two cases with a BSI of 0 or almost 0, the TBU was positive and did change, even though the change was not significant. These findings suggest that the change in TBU is more accurate and sensitive than that in BSI.

We have investigated that BONENAVI^®^ often resulted in false-positive or false-negative in patients with small tumor burden (e.g., EOD = 1 and low activity) [24]. Others have also reported that BSI was strongly influenced by tumor location, volume, and scan uptake time in a simulation study [25] and clinical setting [26]. It could be due to attenuation by scatters and different activity of osteoblasts.

On the other hand, a quantitative analysis using three-dimensional SPECT/CT image improved by three-dimensional correction; attenuation, scatter, depth-dependent resolution, and decay correction [13–15]. This means that problems with planar images can be recovered by SPECT/CT, although the partial volume effect cannot still be ignored.

Of course, the number of patients in the present study was very small; therefore, this should be tested with larger number of patients.

The present study had some limitations. The TBU cut-off value was only obtained using prostate cancer patients. When the TBU is applied to other kinds of cancer patients, similar evaluation should be carried out. Another limitation was the small number of patients who received Ra-223 therapy, and further studies with larger sample sizes are needed to obtain more reliable conclusion. In addition, bone SPECT/CT is essential for determining TBU, which leads to prolongation of the study time and means that retrospective studies are not possible if SPECT of the trunk has not been performed.

In conclusion, we established a new BS evaluation method using three-dimensional bone SPECT/CT. We considered active bone metastatic burden can be obtained by the sum of (uptake value in metastatic lesion) × (lesion volume). We obtained an SUV cut-off value of 7.0 in prostate cancer patients. In this process, we verified that uptake by non-metastatic lesions was lower than in patients with bone metastasis compared with those without bone metastasis. We applied TBU to prostate cancer patients receiving Ra-223 therapy. Despite the small number of patients, change in TBU seemed to have the potential to be more accurate and sensitive than BSI of BONENAVI^®^.
